# Characterization of the gut microbiota in hemodialysis patients with sarcopenia

**DOI:** 10.1007/s11255-021-03056-6

**Published:** 2021-11-29

**Authors:** Qifan Zhou, Hailin Zhang, Lixia Yin, Guilian Li, Wenxue Liang, Guanjie Chen

**Affiliations:** 1 Lianyungang Clinical College of Nanjing Medical University, The First People’s Hospital of Lianyungang, Lianyungang, China; 2 The Affiliated Lianyungang Hospital of Xuzhou Medical University, Lianyungang, China

**Keywords:** Maintenance hemodialysis, Sarcopenia, Gut microbiota, Characterization

## Abstract

**Purpose:**

Maintenance hemodialysis (MHD) patients are at high risk of sarcopenia. Gut microbiota affects host metabolic and may act in the occurrence of sarcopenia importantly. This study aimed to study the characterization of the gut microbiota in MHD patients with sarcopenia, and to further reveal the complex pathophysiology of sarcopenia in MHD patients.

**Methods:**

Fecal samples and clinical data were collected from 30 MHD patients with sarcopenia, and 30 age-and-sex-matched MHD patients without sarcopenia in 1 general hospital of Jiangsu Province from December 2020 to March 2021. 16S rRNA sequencing technology was used to analyze the genetic sequence of the gut microbiota for evaluation of the diversity, species composition, and differential microbiota of the two groups.

**Results:**

Compared to MHD patients without sarcopenia, the ACE index of patients with sarcopenia was lower (*P* = 0.014), and there was a structural difference in the β-diversity between the two groups (*P* = 0.001). At the genus level, the relative abundance of *Tyzzerella_4* in the sarcopenia group was significantly higher than in the non-sarcopenia group (*P* = 0.039), and the relative abundance of *Megamonas* (*P* = 0.004), *Coprococcus_2* (*P* = 0.038), and *uncultured_bacterium_f_Muribaculaceae* (*P* = 0.040) decreased significantly.

**Conclusion:**

The diversity and structure of the gut microbiota of MHD patients with sarcopenia were altered. The occurrence of sarcopenia in MHD patients may be influenced by gut microbiota.

**Supplementary Information:**

The online version contains supplementary material available at 10.1007/s11255-021-03056-6.

## Introduction

Sarcopenia is a group of degenerative syndromes involving the accelerated loss of muscle mass and function [1]. Maintenance hemodialysis (MHD) patients are at high risk of sarcopenia due to negative protein balance caused by kidney disease, dialysis treatment, and chronic inflammation combined with dietary restrictions and reduced physical activity [2, 3]. Sarcopenia reduces the physical function of MHD patients, impairs their ability to conduct daily living activities, and leads to a decline in patients’ quality of life. It also increases the risk of hospitalization and hospitalization costs, and it is an independent predictor of mortality in MHD patients [4–7].

A variety of metabolites produced by the gut microbiota can affect the health of the host [8, 9]. Previous studies have proposed a potential association between the gut microbiota and sarcopenia, forming the "gut-muscle" axis hypothesis [10–12]. It is believed that the gut microbiota affects the muscle mass and function of the host, by directly affecting the host's inflammatory environment [11], bioavailability of nutrients [13], lipid metabolism [14], and the energy supply [15]. Although these have been predominantly demonstrated in animal models, human studies are limited. No study has directly explored the characteristics of the gut microbiota in MHD patients with sarcopenia.

This study aimed to study the characteristics of the gut microbiota of MHD patients with sarcopenia. The results of this study could potentially shed an additional light on the complex pathophysiology of sarcopenia in MHD patients.

## Materials and methods

### Subjects

Convenience sampling was used to evaluate whether patients who received MHD treatment at the dialysis units of one general hospitals in Jiangsu Province, China, from December 2020 to March 2021 met the criteria for inclusion and exclusion. This study complied with the Declaration of Helsinki and was approved by the ethics committees of the hospitals where the study was conducted. Written and informed consent was obtained from all participants of the study.

The inclusion criteria were (1) age ≥ 18 years, (2) ability to communicate normally, (3) hemodialysis for at least 3 months, and (4) dialysis three times a week. Exclusion criteria were (1) taking probiotics, prebiotics antibiotics or laxatives within 3 months before entering the study, (2) inflammatory disease, autoimmune disease, or immunosuppressive treatments, (3) gastrointestinal diseases, diarrhea or constipation, (4) significant changes in diet a week before sampling, (5) serious complications such as liver cirrhosis, hematological diseases, malignant tumor, or serious cardiovascular disease, (6) contraindicated to conduct bioelectric impedance analysis (BIA) test, and (7) history of alcoholism. The control group consisted of MHD patients without sarcopenia, matched for age and sex.

### Diagnosis of sarcopenia

Sarcopenia was diagnosed based on the diagnostic criteria issued by the Asia Working Group for Sarcopenia (AWGS) in 2019 [16]. The criteria included skeletal muscle mass index (SMI), hand grip strength, and 6 m gait speed. A diagnosis of sarcopenia was made when the patient's SMI decreased along with reduced hand grip strength and/or slow gait speed. The diagnostic threshold of each index was as follows: (1) SMI: male < 7 kg/m^2^, female < 5.7 kg/m^2^, (2) grip strength: male < 28.0 kg, female < 18.0 kg, and (3) 6 m gait speed: walking speed < 1 m/s.

### Collection of samples and data

Fresh feces from fecal centers of all subjects were collected in sterile boxes, and stored at − 80 °C immediately after sampling, for subsequent analysis. On the day of fecal collection, venous blood was collected before and after dialysis to assay for hemoglobin, serum albumin, total cholesterol, triglyceride, high-density lipoprotein (HDL), low-density lipoprotein (LDL), and CRP levels, as well as KT/V, which is a measure of the adequacy of dialysis. Demographic and clinical data, such as age, gender, comorbidities, dialysis age, BMI, grip strength, and gait speed were collected through a questionnaire. SMI and fat free mass were measured based on BIA technology (InBodyS10, InBody, China) 30–60 min after dialysis.

### DNA extraction, PCR amplification and 16S rRNA sequencing

DNA was extracted according to the instructions of the NucleoSpin Stool kit, (Macherey Nagel, Germany). DNA concentration and purity were detected using a NanoDrop spectrophotometer, and DNA quality was detected by 1.8% agarose gel electrophoresis. The V3–V4 variable regions of bacterial 16S ribosomal RNA were amplified by PCR with primers 338F and 806R; and the PCR products were mixed in equal proportions, and then purified using a DNA purification column (Omega, American). Sequencing was performed using the Illumina MiSeq platform.

### Bioinformatics analysis

The original sequence was assembled and quality-filtered using Flash 1.2.11 and Trimmomatic 0.33, and the chimera was removed using UCHIME 8.1 software to obtain a valid sequence. USearch 10.0 was used to cluster the sequences at the similarity level of 97% to obtain the operational taxonomic unit (OTU). Diversity was calculated at the OTU level and performed using QIIME2 software. The difference between groups was compared using a *t* test. The ACE index and Chao1 index were used to measure the abundance of species. The Simpson index and Shannon index were used to measure the diversity of species, and the coverage index reflected the sequencing depth of the samples. The β-diversity was calculated according to the Unifrac distance, results of principal coordinate analysis (PCoA), and non-metric multidimensional scaling (NMDS) with R 3.6.1. The rank sum test was used to analyze the markers of gut microbiota between the two groups.

### Statistical analysis

Epidata 3.1 was used for double-enter of the data, and SPSS 26.0 software was used for statistical analysis. Normal measurement data were expressed as $\overline{x} \pm s$, and two independent samples *t* test was used for comparison between the groups. Non-normal distribution data were represented by *M* (*P*_*25*_, *P*_*75*_), and comparisons between groups were made using non-parametric test. Countable data were expressed as percentages, and *χ*^2^ test was used to compare the two groups. Differences were considered statistically significant when *P* < 0.05.

## Result

### General data

There was no statistically significant differences between the MHD patients with or without sarcopenia in terms of age (*P* = 0.215) and gender (*P* = 1). The LDL (*P* = 0.005), BMI (*P* < 0.001), SMI (*P* < 0.001), fat free mass (*P* < 0.001), grip strength (*P* = 0.015), and gait speed (*P* = 0.004) were significantly lower in MHD patients with sarcopenia. Total cholesterol was significantly higher in MHD patients with sarcopenia (*P* = 0.016) (Table 1).

**Table 1:** Comparison of general data between MHD patients with or without sarcopenia

Outcomes	Sarcopenia (*n* = 30)	Non-sarcopenia (*n* = 30)	*P* value
Age(year)	49.9 ± 12.6	45.87 ± 12.3	0.215
Gender(man)	17 (56.7%)	17 (56.7%)	1
Complications			
Diabetes	2 (6.7%)	3 (10.0%)	0.640
Hypertension	20 (66.77%)	17 (56.7%)	0.426
Polycystic kidney	1 (3.3%)	0 (0%)	1
Dialysis age(month)	36 (24.75, 76.25)	35.5 (16.5,65.25)	0.333
Hemoglobin (g/L)	106.23 ± 13.07	114.23 ± 17.99	0.054
Serum albumin (g/L)	41.03 ± 2.625	41.66 ± 3.58	0.329
Total cholesterol (mmol/L)	4.47 ± 0.90	3.90 ± 0.87	0.016
Triglyceride (mmol/L)	1.36 (0.82, 2.20)	1.31 (0.88, 1.72)	0.751
HDL (mmol/L)	1.09 ± 0.29	1.23 ± 0.33	0.093
LDL (mmol/L)	1.97 ± 0.54	2.37 ± 0.53	0.005
CRP (mg/L)	2.61 (1.53, 4.14)	2.92 (1.81, 4.99)	0.290
KT/V	1.53 ± 0.31	1.43 ± 0.29	0.309
BMI (kg/m^2^)	19.93 ± 2.70	24.12 ± 3.94	< 0.001
SMI (kg/m2)	6.07 ± 0.86	8.27 ± 1.60	< 0.001
Fat free mass (kg)	40.50 (31.85, 45.28)	54.00 (41.33, 62.15)	< 0.001
Grip strength (kg)	27.15 (21.25,34.20)	32.25 (27.05,44.60)	0.015
Gait speed (m/s)	0.76 ± 0.24	0.91 ± 0.12	0.004

*HDL* high-density lipoprotein, *LDL* low-density lipoprotein

### Analysis of gut microbiota diversity in MHD patients with or without sarcopenia

**Fig. 1:**
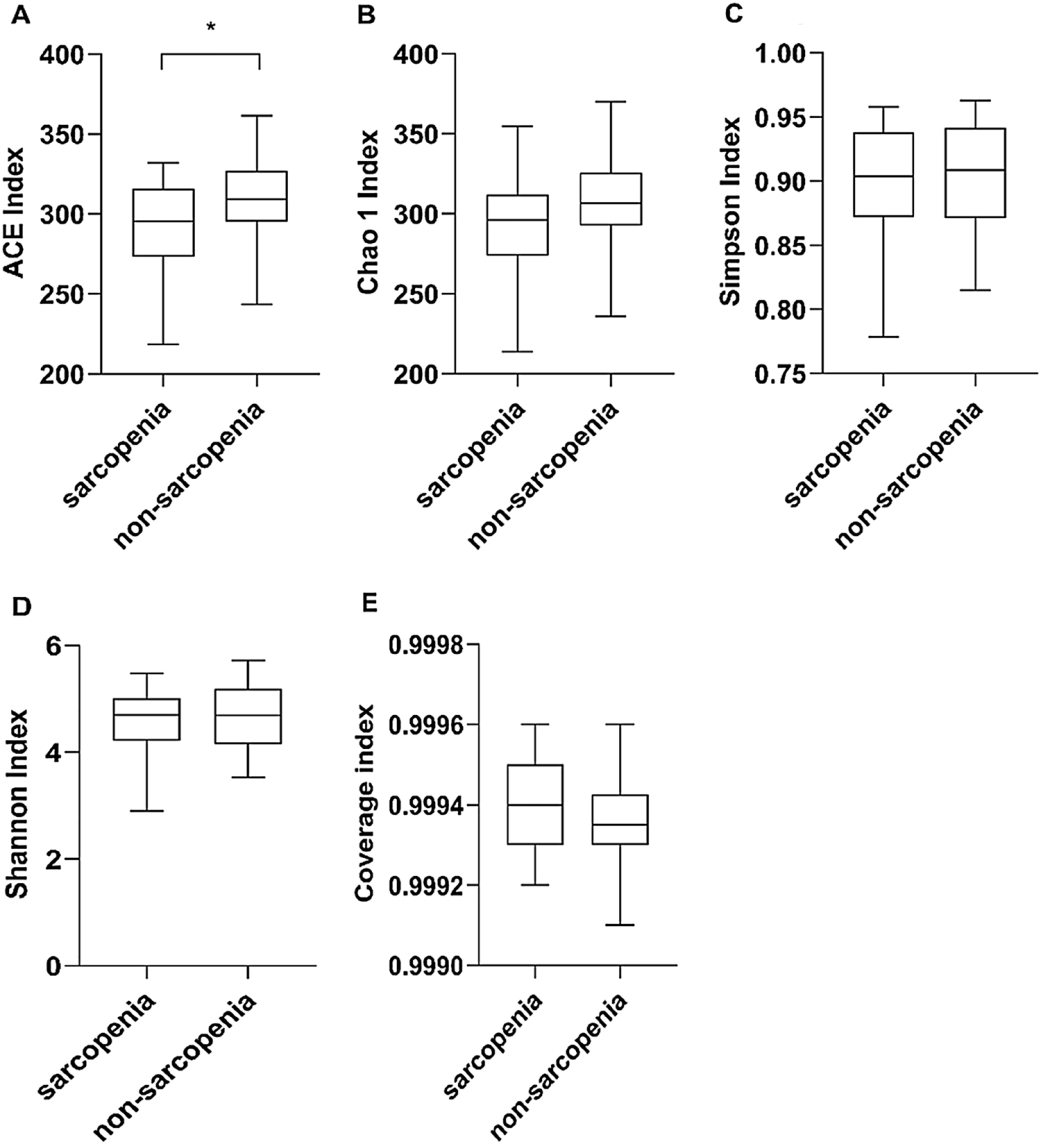
Analysis of gut microbiota α-diversity in MHD patients with or without sarcopenia *P < 0.05

α-Diversity reflects the abundance and diversity of the microflora in a group. The ACE index in the sarcopenia group decreased significantly (*P* = 0.014) (Fig. 1A). The Chao1 (*P* = 0.056), Simpson (*P* = 0.815) and Shannon index (*P* = 0.698) decreased but not significantly in the sarcopenia group (Fig. 1B, C). The coverage index exceeded 99.9%, indicating that the number and sequencing depth of the samples could better reflect the community structure of the groups (Fig. 1E).

**Fig. 2:**
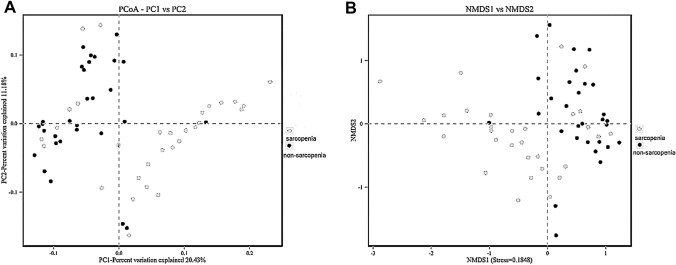
Analysis of β-diversity of gut microbiota in MHD patients with or without sarcopenia

β-Diversity reflected the difference in the composition of the gut microbiota between the two groups. The PCoA diagram showed that PCoA1 and PCoA2 were significantly separated in the sarcopenia and non-sarcopenia groups, accounting for 20.43% and 11.1% of the variation, respectively, along with significant differences in bacterial community compositions (*P* = 0.001) (Fig. 2A). The NMDS graph showed that the difference within the group was small, while the difference between the groups was larger. Stress = 0.1848 < 0.2, implying that the analysis was reliable.

### Comparison of gut microbiota structure and differential microbiota between MHD patients with and without sarcopenia

**Fig. 3:**
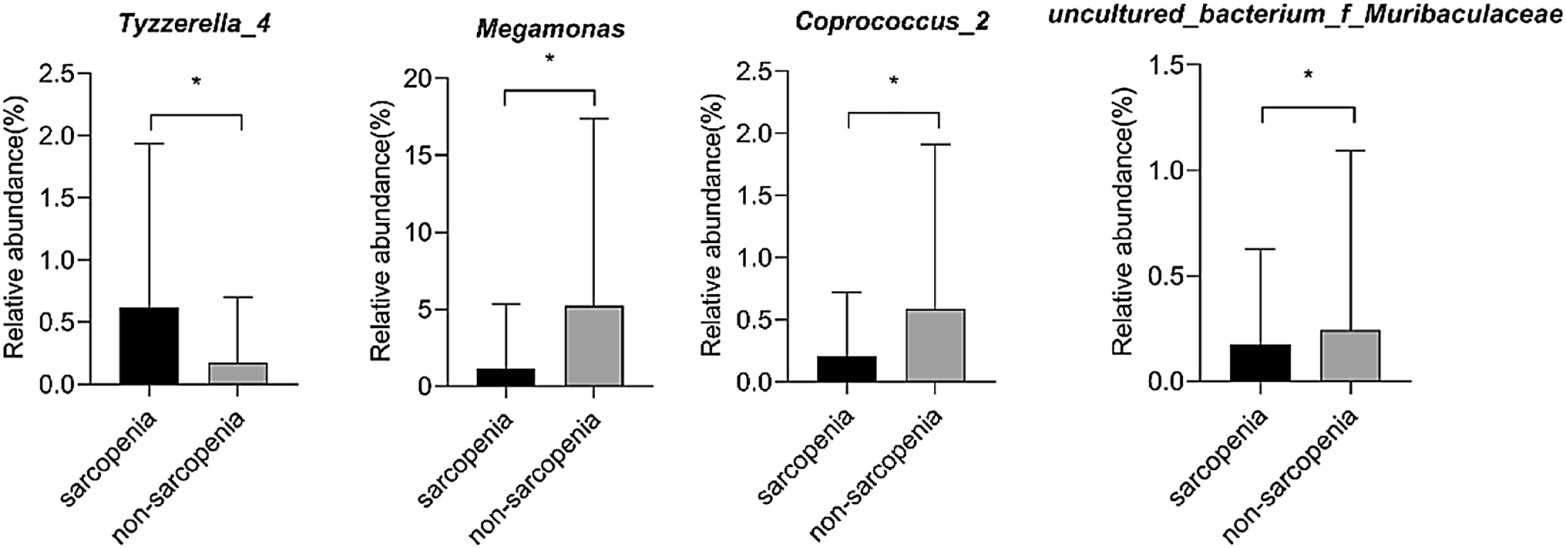
Analysis of gut microbiota at genus level in MHD patients with or without sarcopenia

At the genus level, the most dominant bacteria were *Bacteroides* (sarcopenia, 15.8% vs. non-sarcopenia, 17.9%; *P* = 0.366) and *Faecalibacterium* (sarcopenia 12.9% vs. non-sarcopenia 10.7%; *P* = 0.388). The relative abundances of the four genus were significantly different among the groups. The relative abundance of *Tyzzerella_4* in the sarcopenia group was significantly higher (*P* = 0.039), and the relative abundance of *Megamonas* (*P* = 0.004), *Coprococcus_2* (*P* = 0.038), and *uncultured_bacterium_f_Muribaculaceae* (*P* = 0.040), were significantly lower in the sarcopenia group (Fig. 3).

## Discussion

The gut microbiota has become the forefront of exploring pathophysiology related to nutritional status and disease states. In this study, 16S rRNA sequencing technology was used to analyze the characteristics of gut microbiota in MHD patients with sarcopenia. The results showed that MHD patients with sarcopenia had lower ACE index compared with MHD patients with non- sarcopenia, and there was a structural difference in the β-diversity between the two groups. In terms of gut microbiota composition, 4 genera were significantly different between the two groups.

This study found that the diversity of the gut microbiota in the sarcopenia group was significantly lower than that of the non-sarcopenia group, and the difference in the structure of the gut microbiota between the two groups was significant. Previous studies have showed that a stable and diverse gut microbiota is beneficial for the quality and function of skeletal muscle. It was reported that the expression of insulin-like growth factor-1, gene transcription related to skeletal muscle growth, and mitochondrial function significantly decreased leading to skeletal muscle atrophy in germ-free mice, when compared to normal mice colonized by bacteria. After transplanting the gut microbiota of mice with bacteria into germ-free mice, the skeletal muscle mass of the sterile mice increased significantly [17]. In an RCT study, prebiotic supplementation significantly improved the grip strength in frail elderly people [18]. Diversified and stable gut microbiotas dominated by beneficial bacteria can affect energy metabolism through the metabolites produced, thereby improving body composition [19]. Data from this and previous studies, thus, suggest that sarcopenia is closely related to the diversity of the gut microbiota. Decreased diversity and structural instability of the gut microbiota may lead to sarcopenia in MHD patients.

The results of this study showed that compared with MHD patients without sarcopenia, *Tyzzerella_4* was significantly higher in MHD patients with sarcopenia while *Megamonas*, *Coprococcus_2*, and *Uncultured_bacterium_f_muribaculaceae* were significantly lower.

*Tyzzerella_4* is a potential pathogen associated with a poor diet and obesity. In this study, the relative abundance of *Tyzzerella_4* in MHD patients with sarcopenia was significantly higher, which may be related to the high-fat diet of MHD patients. Previous studies have found that the relative abundance of *Tyzzerella_4* was significantly higher in mice fed a high-fat diet [20]. Similar trends were observed in obese people and people consuming unhealthy diets [21, 22]. A high-fat diet increases body fat tissue, and it is associated with thinner skeletal muscle fibers, increased proteolysis and inflammation in muscle tissue, as well as decreased muscle strength [23, 24]. This may be the influencing factor of *Tyzzerella_4* in sarcopenia. Therefore, the gut microbiota may play an important role in the reduction of muscle mass and strength in MHD patients due to diet.

In this study, the abundance of *Megamonas* and *Coprococcus_2* were significantly lower in the sarcopenia group. There are few studies on *Megamonas* in the sarcopenia population, but many other studies have shown that the relative abundance of *Megamonas* is significantly reduced in people with low BMI [25–28]. A low BMI indicates malnutrition, and it can easily result in sarcopenia [29]. A previous study showed that *Coprococcus_2* was negatively correlated with the frailty index [30], while frailty and sarcopenia were both caused by the decline of multi-system functions such as in aging-related loss of physical functions [31]. This may be because both *Megamonas* and *Coprococcus_2* belong to firmicutes that can produce short chain fatty acids (SCFAs) [32, 33]. SCFAs are products of cellulose, hemicellulose, and other substances that cannot be directly digested by gastric digestive enzymes, which are subsequently digested and fermented by the gut microbiota. They have a variety of beneficial effects on muscle quality and function. SCFAs not only improve the permeability of the gut microbiota, but also regulate glucose uptake, reduce insulin resistance and fatty acid oxidation, affect energy supply, plus even regulate immunity and inhibit the secretion of inflammatory response factors [34]. Animal experiments have also directly confirmed the beneficial effects of SCFAs on muscles. Skeletal muscle injury was partially reversed in germ-free mice fed with SCFA [17], and mice with reduced exercise capacity were significantly improved after SCFA supplementation [35]. Therefore, gut microbiota may affect the sarcopenia in MHD patients by manipulating the production of SCFA.

In this study, the abundance of *uncultured_bacterium_f_muribaculaceae* in the sarcopenia group was significantly reduced. A previous study found that *uncultured_Bacterium_F_Muribaculaceae* was negatively correlated with blood lipid levels [36]. Blood lipids can affect the fatty acid composition of the cell membrane, causing chronic inflammation and insulin resistance. Chronic inflammation promotes protein decomposition, and insulin resistance affects the energy supplies of muscle cells, which eventually leads to a decline in muscle mass and strength [37]. However, there are few studies on *uncultured_bacterium_F_Muribaculaceae*, and further research is needed to understand its role in the pathophysiology of sarcopenia in MHD patients.

This study had several limitations. First, this was a cross-sectional study with a small sample size and could not confirm the causal relationship between the gut microbiota and sarcopenia in MHD patients. Secondly, this study only collected sample data from one local region, and the extension of the findings requires further research encompassing a wider region. Finally, this study used 16S rRNA amplicon sequencing technology, which limits further research on bacterial colony identification, gene prediction, and functional annotation.

This study showed that the diversity and structure of the gut microbiota in MHD patients with sarcopenia was significantly different from those in MHD patients without sarcopenia, and potentially shed an additional light on the complex pathophysiology of sarcopenia in patients on MHD. A large-sample multicenter interventional study, based on metagenomic sequencing, is needed to establish causal relationships, determine the functional pathways between the gut microbiota and sarcopenia in MHD patients.

## Supplementary Information

Below is the link to the electronic supplementary material.

Supplementary file1 (PDF 7928 KB)

Supplementary file2 (XLXS 22 KB)

Supplementary file2 (JPEG 441 KB)
