# Supplementary material for: Characterization of the gut microbiota in hemodialysis patients with sarcopenia
Source: Int Urol Nephrol. 2021 Nov 29;54(8):1899–906. doi: 10.1007/s11255-021-03056-6 (PMC9262794; doi:10.1007/s11255-021-03056-6)
Supplement: Supplementary file 1 — Supplementary file1 (PDF 7928 KB) [file 11255_2021_3056_MOESM1_ESM.pdf]

连云港市卫生健康委员会文件

连卫老健〔2021〕5号

关于公布2021年度连云港市老龄健康科研项目评审结果的通知

各县区卫生健康委，委直属各有关医疗机构:

根据《关于组织申报2021年度连云港市老龄健康科研项目的通知》（连卫办老健〔2021〕3号），我委组织开展了2021年度市老龄健康科研项目评审工作。根据评审结果，确认“基于ERAS理念的多学科协作诊疗模式在老年骨质疏松骨折的临床应用研究”等15个项目为2021年老龄健康面上科研项目、“3D打印联合经皮椎骨成形术治疗骨质疏松性Kummell病的临床疗效分析”等5个项目为老龄健康指导性科研项目（具体各类项目立项情况见附件），现将有关事项通知如下:

一、各项目单位要高度重视老龄健康科研项目建设工作，面

上科研项目按照资助经费不低于1:1的比例安排配套经费，做到足额到位、严格管理、专款专用，保证项目所需；指导性科研项目经费由项目单位自筹，各单位要为项目负责人提供必要的条件和经费支持。同时，积极推动科研成果应用转化，加快提升老龄健康服务水平，为提高全市老年人的健康水平做出贡献。

二、各单位要严格履行合同，认真组织项目实施，加强过程管理，为项目实施提供必要的条件，确保按时、优质完成科研项目。

三、本次评审确认的所有项目，执行年限均为2022年1月1日至2024年12月31日，需与我委签订《连云港市老龄健康科研项目合同书》，项目主研人登陆市卫健委官网下载专栏下载，同时纸质合同书一式三份于11月20日前报送委老龄健康处。寄送地址：海州区迎曙路66号市公共卫生中心1606室；联系人：王晶晶；电话：80680735。

附件：1.2021年度市老龄健康面上科研项目一览表
2.2021年度市老龄健康指导性科研项目一览表

连云港市卫生健康委员会
2021年11月1日

（信息公开形式：主动公开）

连云港市卫生健康委员会办公室　　2021年11月1日印发

附件 1

2021 年度市老龄健康面上科研项目一览表

项目编号	项目名称	承担单位	项目负责人	经费
L202101	基于 ERAS 理念的多学科协作诊疗模式在老年骨质疏松骨折的临床应用研究	连云港市第一人民医院	张艳艳	1 万元
L202102	ctDNA 甲基化谱无创检测技术在老龄前列腺癌患者早期诊断中应用转化研究	连云港市第一人民医院	薛鹏	1 万元
L202103	肠道菌群在老年维持性血液透析患者肌少症中的早期识别价值	连云港市第一人民医院	张海林	1 万元
L202104	老年患者 CEA 术后颈内动脉平均血流速度的变化及其对脑血流动力学及脑灌注的影响	连云港市第一人民医院	张洪伟	1 万元
L202105	基于双能 X 线探讨 LncRNA- H19 在老年糖尿病性骨质疏松的临床应用价值	连云港市第一人民医院	王国凤	1 万元
L202106	镜像神经元系统对阿尔兹海默病患者认知及言语障碍的影响	连云港市第一人民医院	蒋孝翠	1 万元
L202107	4R 危机管理理论在预防老年住院患者跌倒中的应用研究	连云港市第一人民医院	徐艳	1 万元
L202108	双 PLD 3D-ASL 技术在血管性认知障碍脑血流动力学分析及预后评估中的应用研究	连云港市中医院	周建国	1 万元
L202109	探索智慧医疗+医疗养老服务机构签约合作的医养结合服务模式改善衰弱老人综合评估的研究	连云港市第二人民医院	董燕	1 万元
L202110	医养结合视角下智慧护理对中风患者预后的影响	连云港市第二人民医院	刘瑞凤	1 万元
L202111	类风湿关节炎合并骨质疏松老年患者健康促进方案的构建及应用研究	连云港市第一人民医院	薛艳艳	1 万元
L202112	幸福科学伦理下热情照顾在医养结合养老机构尿失禁老年人中的应用研究	连云港市第一人民医院	高静	1 万元
L202113	精神运动康复干预治疗应用于老年认知衰弱患者中的临床研究	连云港市第一人民医院	张伟伟	1 万元
L202114	智慧养老模式下功能性胃肠病老年患者健康管理体系的构建及应用	连云港市第一人民医院	管振华	1 万元
L202115	托法替布对老年类风湿关节炎伴发骨质疏松患者骨密度的影响	连云港市第一人民医院	邵平	1 万元

附件 2

2021 年度市老龄健康指导性科研项目一览表

项目编号	项目名称	承担单位	项目负责人
L202116	3D 打印联合经皮椎骨成形术治疗骨质疏松性 Kummell 病的临床疗效分析	连云港市第二人民医院	吕南宁
L202117	长针骶刺配合焦氏头针治疗老年中重度膀胱过度活动症随机对照研究	连云港市中医院	姚文平
L202118	低剂量 CT 头颈血管成像与高场磁共振血管成像对老年头颈部动脉粥样硬化狭窄的诊断价值研究	灌南县第一人民医院	吉廷举
L202119	中药抗骨质疏松对老年卒中患者运动障碍恢复的影响	灌云县中医院	骆亚芹
L202120	快速康复外科理念治疗老年骨质疏松股骨粗隆间骨折的临床研究	灌云县人民医院	牛海明
