# Supplementary figures and images for: Characterization of the gut microbiota in hemodialysis patients with sarcopenia
Source: Int Urol Nephrol. 2021 Nov 29;54(8):1899–906. doi: 10.1007/s11255-021-03056-6 (PMC9262794; doi:10.1007/s11255-021-03056-6)

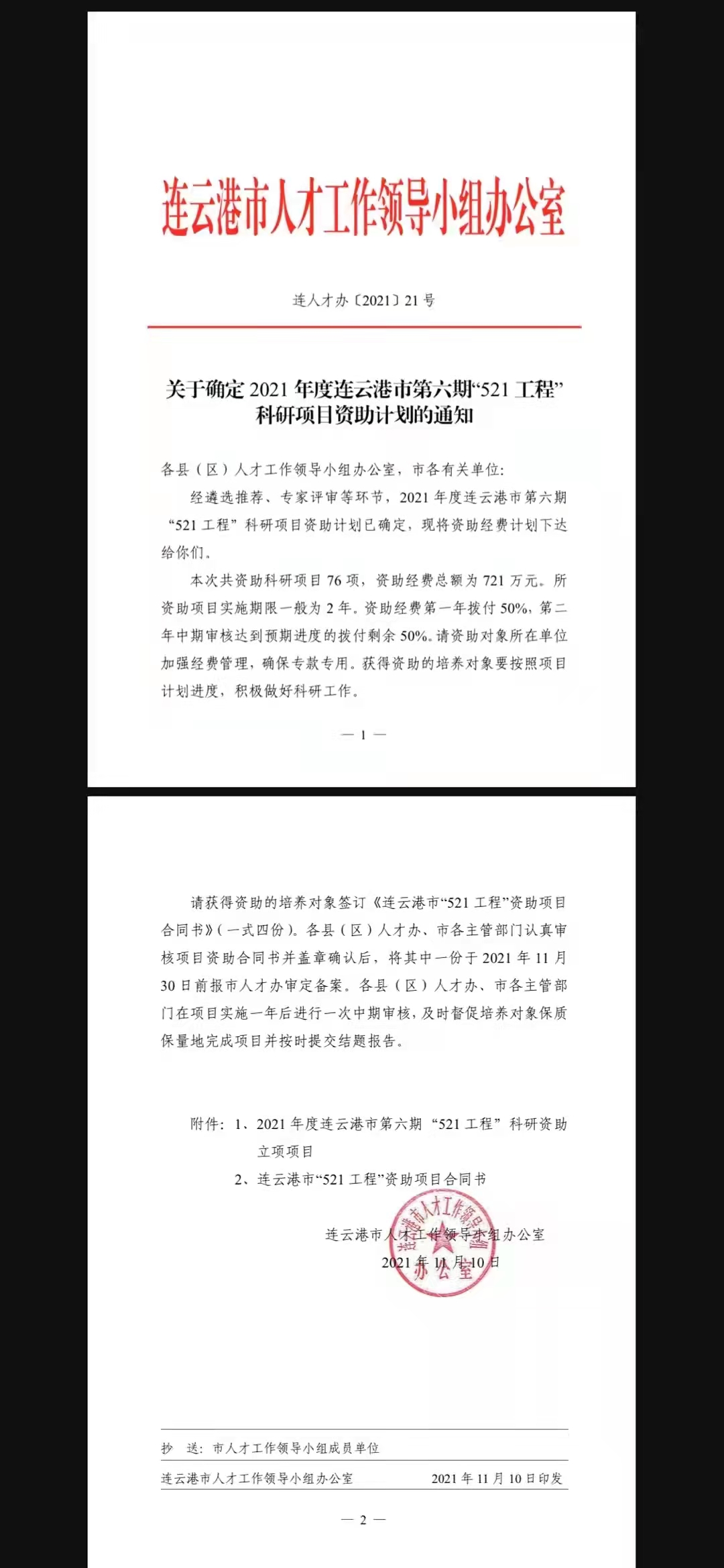

Supplement: Supplementary file 3 — Supplementary file2 (JPEG 441 KB) [file 11255_2021_3056_MOESM3_ESM.jpg]
